# Boosting projection contrast method for co-aperture infrared optical engine design

**DOI:** 10.1016/j.isci.2026.116066

**Published:** 2026-05-22

**Authors:** Yue Pan, Zhenyu Wang, Xiping Xu, Liang Xu, Yongning Zhang, Saisai Sun, Motong Hu

**Affiliations:** 1 School of Optoelectronic Engineering, Changchun University of Science and Technology, Changchun, Jilin 130022, China; 2 Xi’an Institute of Optics and Precision Mechanics, Chinese Academy of Science, Xi’an, Shanxi 710119, China; 3 National Key Laboratory of Electromagnetic Space Security, Tianjin 300308, China

**Keywords:** applied sciences, engineering

## Abstract

The diffraction characteristic of digital micromirror device (DMD) is the main factor to reduce the projection contrast, making it incompetent for DMD-based infrared scene projector (IRSP) to evaluate and test advanced infrared imaging systems in the hardware-in-the-loop (HWIL) simulation. In this paper, the diffraction analysis is applied for boosting the projection contrast of a dual-DMD based infrared optical engine. The diffraction directions of different orders are accurately calculated to maximize the DMD optical efficiency. Meanwhile, a two-element achromatic total internal reflection (ATIR) prism group is optimized to ensure the optimal illumination incident angle derived from the diffraction calculation. Furthermore, the diffracted beams of second maximums under off-state micromirrors are traced to demonstrate that irradiation distribution is close to the level without considering diffraction. Ultimately, these simulation results are validated by an experimental platform. The boosting contrast method can also be popularized to design other DMD-based optical instruments.

## Highlights

• A diffraction guided design method was proposed to improve projection contrast
• Developed is an optimized ATIR prism group to match the diffraction-optimized illumination
• Verified the feasibility of the infrared dual channel common aperture optical engine

## Graphical abstract

**Figure:**
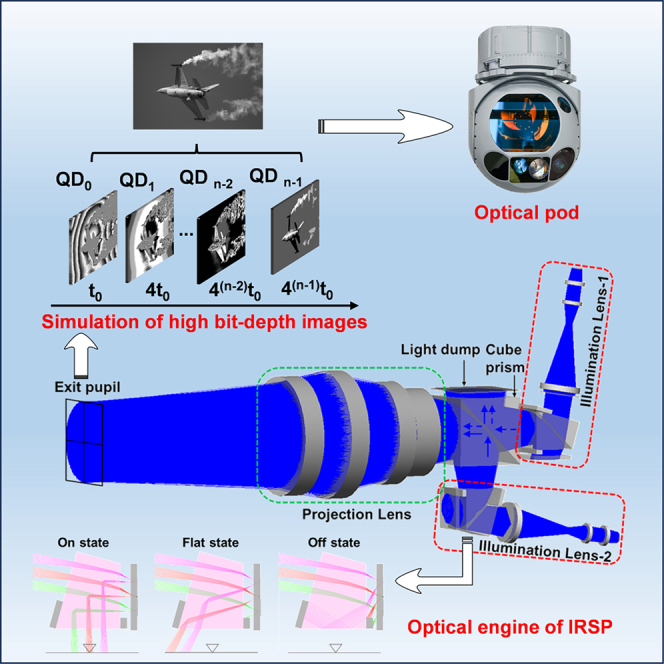

## Introduction

Infrared scene projector (IRSP) can project virtual infrared scenes with radiation characteristics conforming to real scenes in the hardware-in-the-loop (HWIL) simulation, which enables the performance evaluation of infrared imaging systems in target tracking and recognition.^1^^,^^2^^,^^3^^,^^4^ As a testing device, the projection dynamic range of IRSP should be superior to the capture ability of advanced infrared imaging systems. A series of methods have been proposed to enhance the projection bit-depth, which describes how many levels the projected energy range from the minimum to the maximum is uniformly divided into.^5^^,^^6^^,^^7^ While the range width of the projected energy depends on the projection contrast of the optimized optical engine. We once utilized two digital micromirror devices (DMDs) in parallel to achieve ultra-high bit-depth for visible projection.^8^^,^^9^ In order to promote the technology to the development of IRSP, a dual-DMD based infrared optical engine with high contrast should be designed to exhibit the advantages of high bit-depth and realize the high dynamic range (HDR) infrared projection.

Customarily, the configuration of optical engine is determined by infrared scene generation devices (IRSGDs), such as laser diode arrays, resistive arrays, blackbody microcavity arrays, infrared liquid crystal on silicon (LCoS), and digital micromirror device (DMD), where DMD is the most widely used as a reflective spatial light modulator with the feature of polarization insensitive.^10^^,^^11^^,^^12^^,^^13^ The first is the traditional DMD, where the micromirror can only be tilted by ±12° diagonally in one direction.^14^^,^^15^ The other is the tilted rolling pixel (TRP) DMD, where the micromirror tilts ±12° diagonally in both directions, namely pitch rotation and roll rotation, to achieve a micromirror tilt of ±17°. The larger tilt angle increases the illumination cone angle of the DMD active area, thereby expanding the optical Étendue and improving the illumination efficiency.

Owing to the special illumination angle of DMD, the total internal reflection (TIR) prism is usually used to separate the illumination and projection light paths corresponding to different states of the micromirror. In the visible band, Pan et al.^16^ designed a three-element TIR prism group for the traditional DMD. The modulated beam of the DMD is initially totally reflected by the prism to the DMD active area. It prevents the energy of the micromirrors in the off-state and flat-state from entering the projection lens to form stray light. In addition, they designed a two-element achromatic TIR (ATIR) prism, where the beam is first transmitted to the DMD active area and then totally reflected to the projection lens, significantly reducing the influence of lateral chromatic aberration on illumination uniformity.^17^^,^^18^^,^^19^ The ATIR prism will amplify the projected cone angle in angular and object space, enabling the flat-state energy to enter the projection lens. This problem can be solved by placing a baffle at the illumination aperture stop or by combining an X-Y polynomial lens with an ATIR prism.^20^^,^^21^ In addition, the illumination beam passing through the prism will be incident on the TRP DMD active area at an unacceptably large angle, which results in spot distortion. Therefore, Bai et al.^22^ proposed a non-coaxial illumination based on the Scheimpflug principle to improve the illumination uniformity.

The maximum pixel pitch of the traditional DMD is 13.68 μm, and the maximum pixel pitch of the TRP-DMD is only 5.4 μm. It is generally believed that the larger the pixel pitch, the weaker the diffraction effect. For this reason, the traditional DMD is used to design the IRSP. Qiao et al.^23^ designed a long-wave IRSP based on a three-element TIR prism group, which has the same function with the one designed by Jui-Wen Pan. Du et al.^24^ constructed the constraint model and optimized the TIR prism group, which further improved the contrast of the mid-wave infrared (MWIR) projection image. To further expand the simulation ability of IRSP, the co-aperture optical engine with two channels is usually adopted to combine dual DMDs in parallel. Pan et al.^25^^,^^26^ utilized dual DMDs to generate MWIR and long-wave infrared (LWIR) scene images, respectively, thereby simulating the differences in energy distribution of different bands. To reduce the design difficulty of the optical engine, they adopted the mode of direct illumination and separated on-state and off-state beams with the spatial stereo layout. Such a configuration can only alleviate the mutual restraint between the projection and the illumination by increasing the back working distance, further rising the difficulty of assembly and alignment. Moreover, on-state and flat-state beams cannot be effectively separated.

In the infrared band, the DMD diffraction causes the principal maximum (PM) direction to deviate from the projection axis and the adjacent secondary maximum (SM) beams to enter the projection lens, severely limiting the projection quality of IRSP.^27^^,^^28^ To weaken the diffraction influence, a diffraction model is typically established to predict the energy distribution. Qiang et al.^29^ regarded DMD as a one-dimensional diffraction grating to compensate for the angular dispersion introduced by the DMD. However, the lack of dimensions causes the diffraction model to be inconsistent with the physical properties of DMD.

To improve the optical efficiency of projection lithography under 343 nm laser wavelength, Deng et al.^30^ investigated the effects of pixel pitch, operating wavelength, and incident angle on the PM diffraction efficiency. Considering that the scalar diffraction model is invalid in the LWIR band, Han et al.^31^^,^^32^ established a vector diffraction model and verified that IRSP has the best projection quality when the incident azimuth angle is 0° and the elevation angle is between 44° and 48°. Wang Chao et al.^33^ investigated the impact of diffraction angle changes on polarization distortion to improve the polarized beam splitter. Du et al.^34^ also constructed the vector diffraction model to design the illumination system of DMD in the LWIR band.

The current research mainly improves the PM efficiency but ignores the consistency between the PM direction and the projection axis. Thereby, the PM directions of some wavelengths may deviate too much from the projection axis, causing the SM beams to enter the projection lens and reduce the projection quality. Especially for dual-DMD based IRSPs, existing research often lacks analysis and effective suppression of SM beams from both on-state and off-state DMD. Such stray light elevates the lower limit of projection contrast, thereby directly degrading the overall contrast performance. In order to solve the diffraction problem and boost the projection contrast of the co-aperture optical engine in the MWIR band, we optimize the optical configuration via quantitatively calculating the relationships among wavelength, incident direction, pixel pitch, and directions of each diffraction order. Meanwhile, a two-element ATIR prism group is optimized to guarantee the derived illumination incident angle. Through combining a co-aperture projection lens with two illumination lenses, we ultimately make up the dual-DMD based infrared optical engine. And the image quality under the diffraction influence is also discussed in detail. The findings of this study can also be popularized to design other DMD-based optical instruments.

The rest of the article is arranged as follows: firstly, the diffraction characteristics of DMD and the optimal illumination angle are discussed. Secondly, according to diffraction analysis, the achromatic prism, illumination and projection lens of the optical engine are optimized respectively. Finally, simulation analysis and experimental verification prove that the proposed method can mitigate the degradation of projection quality caused by diffraction effects.

## Results

### Diffraction characteristic analysis of DMD

**Figure 1:**
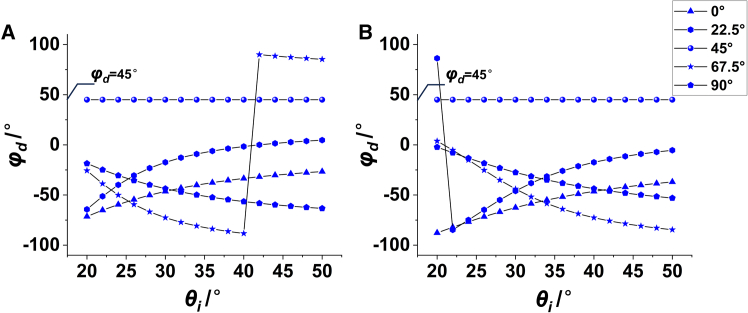
Relationship between the incident direction (*θ*_i_, *φ*_i_) and the azimuth angle *φ*_d_, DMD pixel pitch d = 13.68 μm (A) λ = 3.5 μm. (B) λ = 4.0 μm.

DMD can be regarded as a two-dimensional diffraction grating, which can be quantitatively described as^35^^,^^36^

(Equation 1) $$\left\{ \begin{array}{l} \sin\;\theta_{d}\;\cos\;\varphi_{d}=\sin\;\theta_{i}\;\cos\;\varphi_{i}+p\lambda /d\\ \sin\;\theta_{d}\;\sin\;\varphi_{d}=\sin\;\theta_{i}\;\sin\;\varphi_{i}+q\lambda /d \end{array}\right.\text{,}$$

where the incident elevation angle *θ*_i_ and incident azimuth angle *φ*_i_ express the direction of the illumination beam, the diffraction elevation angle *θ*_d_ and diffraction azimuth angle *φ*_d_ express the direction of the diffraction beam, *λ* is the wave length, *d* is the pixel pitch of micromirror, and (*p*, *q*) represents the diffraction order. According to Equation 1, the diffraction direction of each order can be determined and the corresponding diffraction efficiency is calculated as^37^^,^^38^

(Equation 2) $$\eta =\frac{I}{I_{0}}={\text{sinc}}^{2}\left(p+d\cdot \xi /\sqrt{2}\right){\text{sinc}}^{2}\left(q+d\cdot \xi /\sqrt{2}\right)\text{,}$$

where $\xi =1/\lambda \;\tan\;\gamma \left(\cos\;\theta_{i}+\cos\;\theta_{d}\right)$ is the spatial frequency shift caused by the flipped micromirror, and *γ* is the tilt angle of micromirror. The order with the highest diffraction efficiency is defined as the PM. We performed a quantitative diffraction analysis on the DMD with 1024 × 768 resolution and 13.68 μm pixel pitch, and 3.5 μm and 4.0 μm were selected as the incident wavelengths. According to Equation 1 and Equation 2, when *p* = *q* = −1, the diffraction efficiency is the highest, namely the PM. When *φ*_i_ is set to different values, *θ*_i_ is changed to solve the *φ*_d_ of PM. The result is shown in Figure 1, which shows that the PM direction is concentrated at a stable azimuth angle when the incident azimuth is 45°.

**Figure 2:**
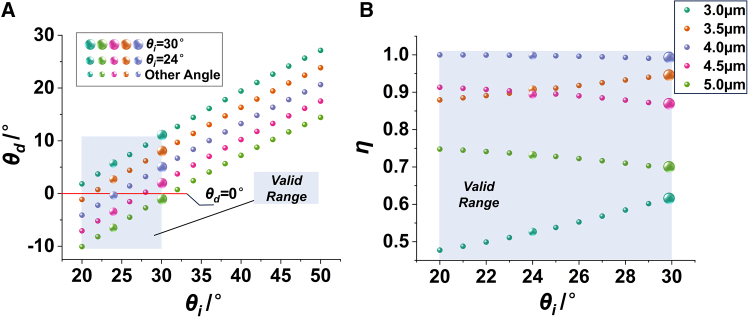
Diffraction distribution within the band of 3 μm–5 μm, DMD pixel pitch d = 13.68 μm (A) Relationship between *θ*_i_ and *θ*_d_. (B) Relationship between *θ*_i_ and *η.*

By changing *θ*_i_, the *θ*_d_, efficiency of PM can be solved. Figure 2 shows the calculated results for incident wavelengths of 3 μm, 3.5 μm, 4.0 μm, 4.5 μm, and 5.0 μm. As shown in Figure 2A, *θ*_d_ increases as *θ*_i_ increases. When *θ*_d_ = 0°, the PM direction coincides with the projection axis. When *θ*_i_ is greater than 30°, the PM direction of any wavelength in the range of 3 μm–5 μm deviates from the projection axis. Thus, the 20°–30° interval is defined as the effective interval. In addition, Figure 2A shows that the energy of different wavelengths cannot completely coincide at the projected exit pupil position. This dispersion caused by diffraction significantly reduces the edge sharpness, which is directly observed by receiving the projection image with the thermal imager. This is one of the reasons why the quality of infrared projection images is worse than that of visible projection images.

In the infrared band, the minimum value of PM efficiency of a single wavelength is usually taken as the DMD diffraction efficiency. Figure 2B shows that for any *θ*_i_ in the effective region, the corresponding minimum value is between 48% and 62%. When *θ*_i_ = 30°, *η* is greater than 62% at any operating wavelength. However, when *θ*_i_ = 24°, *η* of any operating wavelength is only greater than 52%. Meanwhile, the angle between the SM beams and the projection axis is small, which makes it easier to enter the projection lens. For example, when *p* = *q* = 0, the direction of the diffraction beam follows the reflection law, and *θ*_d_ is equal to *θ*_i_. Therefore, *θ*_i_ should be increased as much as possible so long as the diffraction efficiency is close.

**Figure 3:**
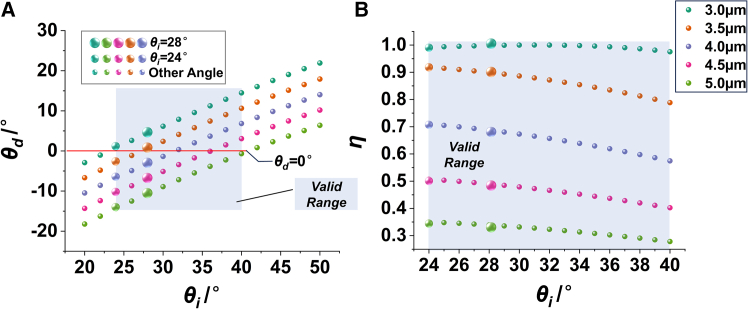
Diffraction distribution within the band of 3 μm–5 μm, DMD pixel pitch d = 10.8 μm (A) Relationship between *θ*_i_ and *θ*_d_. (B) Relationship between *θ*_i_ and *η*.

With other conditions unchanged, modify the DMD pixel pitch to *d* = 10.8 μm. It can be seen from Figure 3A that the effective range is 24°–40°. Figure 3B shows that, within the effective range, the minimum value of diffraction efficiency is close to 30%, which is much lower than the case where the pixel pitch is 13.68 μm.

In summary, to minimize diffraction stray light and achieve optimal MWIR projection quality, the recommended configuration employs a DMD with a 13.68 μm pixel pitch at a 30° incident elevation angle.

### Co-aperture infrared optical engine

**Figure 4:**
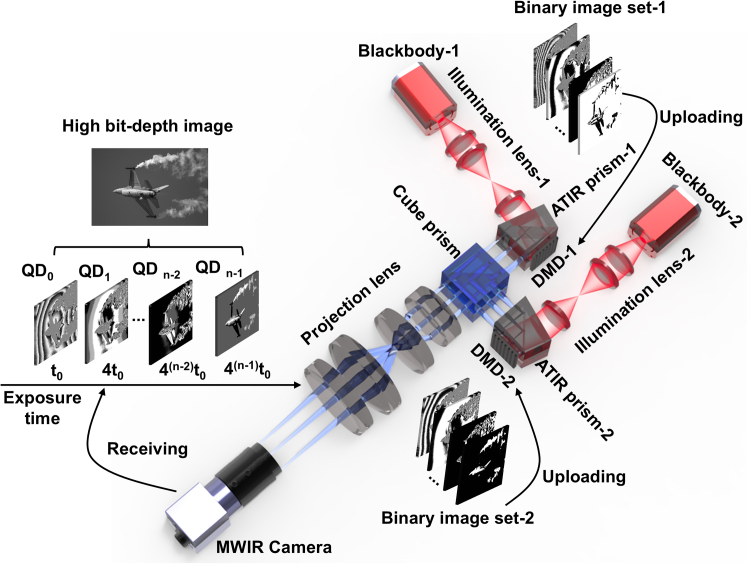
Working principle of the dual DMD-based IRSP

The working principle of the co-aperture infrared optical engine is shown in Figure 4. The beam emitted by the blackbody is shaped and homogenized by the illumination lens before entering the ATIR prism. And the illumination energy of two DMDs should guarantee that the projected energy of one DMD is twice that of the other. Before projection, a high bit-depth image is initially decomposed into a set of quaternary digit-planes (QDs), namely QD decomposition (QDD). Then each QD is further decomposed into two binary images through binary decomposition (BD). When the two binary images are uploaded simultaneously to the two synchronized DMDs, which project and completely overlap the images on the detector of the MWIR camera, namely projecting a QD. Similarly, all QDs must be sequentially projected, from the least significant QD_0_ to the most significant QD_n-1_. In this process, the quaternary pulse width modulation (QPWM) is used to control the exposure time of each QD to guarantee that the exposure time of the next QD is four times that of the previous one. When all the QDs are projected and received by the detector, the MWIR camera can obtain the high bit-depth image. This method leverages the co-aperture optical engine to combine two DMDs in parallel, making up a quaternary spatial light modulator driven by the QPWM to improve the grayscale modulation efficiency for high bit-depth images. Compared with the traditional binary pulse width modulation (BPWM) based on a single DMD, the projection frame rate of the high bit-depth image can be improved by three times. Nevertheless, the optical engine must provide high projection contrast to exhibit the advantages of high bit-depth. Thus, the diffraction model is introduced to optimize the optical engine in the MWIR band. And the specific parameters of optical engine are shown in Table 1.

**Table 1:** Parameters of the optical engine

Parameter	Value
Operating band	3 μm −5 μm
Field of view	±3.5°
F/#	2.34
Back working distance	100 mm
Exit pupil distance	150 mm
Aperture	60 mm
DMD active area	14.1 mm × 10.5 mm
Pixel pitch	13.68 μm
Window glass	sapphire
Blackbody aperture	8 mm

### Illumination optimization

**Figure 5:**
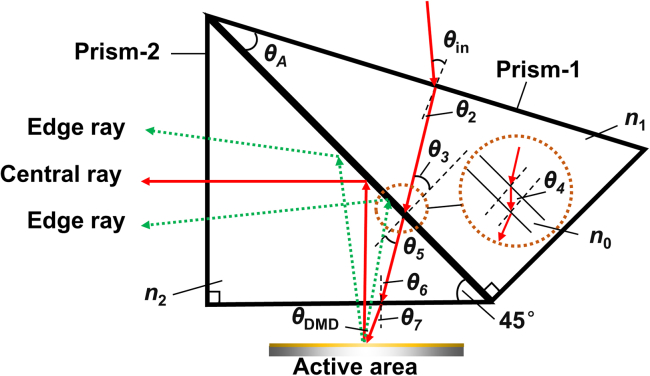
Schematic of optical path in ATIR

The schematic of optical path in ATIR is shown in Figure 5. The modulated beam should be incident on the prism-2 total reflection surface along the normal of the DMD active area and emitted along the projection axis, thus, prism-2 is an isosceles right triangle. To ensure that the incident elevation angle θ_DMD_ is 30°, the relationship between the prism angle θ_A_ and the incident angle θin is solved in prism-1.

According to the geometric relationship and Snell’s law

(Equation 3) $$\sin\;\theta_{\text{in}}=n_{1}\;\sin\left\{\arcsin\left\{n_{2}/n_{1}\;\sin\left[45{}^{\circ}-\arcsin\left(\sin\;\theta_{\text{DMD}}/n_{2}\right)\right]\right\}-\theta_{A}\right\}\text{,}$$

where *n*_1_ and *n*_2_ are the refractive indices of the two prisms, respectively. *θ*_3_ should be less than the critical angle of prism-1 to allow the illumination beam entirely pass through the air gap and enter prism-2. Thus,

(Equation 4) $$\theta_{3}=\arcsin\left\{n_{2}/n_{1}\;\sin\left[45{}^{\circ}-\arcsin\left(\sin\;\theta_{DMD}/n_{2}\right)\right]\right\}<\arcsin\left(1/n_{1}\right)\text{.}$$

The maximum cone angle of the modulated beam is 12°, and the angle deflection of the edge ray after entering prism-2 is arcsin (sin12°/*n*_2_). In addition to the central ray, the incident angles of edge rays on the total reflection surface should also be greater than the critical angle of prism-2, namely

(Equation 5) $$45^{\circ }\pm \arcsin\left(\sin\;12^{\circ }/n_{2}\right)>\arcsin\left(1/n_{2}\right)\text{.}$$

According to Equations 4 and 5, the refractive index range of prism-2 is 1.64 < *n*_2_ < 1.97. Therefore, PbF_2_ with a refractive index of 1.70–1.72 in the MWIR band was selected as the material of prism-2. The refractive index of infrared materials and the calculation results of the corresponding critical angle and *θ*_3_ in prism-1 are shown in Table 2. When prism-1 is made of infrared materials with larger refractive index such as Ge, ZnSe, and ZnS, *θ*_3_ is close to the critical angle, which is not conducive to the illumination optimization. While BaF_2_ and CaF_2_ exhibit similar optical properties in the infrared band, CaF_2_ has higher mechanical hardness and lower hygroscopicity Therefore, PbF_2_ and CaF_2_ were selected as the materials for prism-1.

**Table 2:** Refractive index of common infrared materials and associated angle of prism-1

Material for first prism	Refractive index	Critical angle	*θ*_3_
Ge	4.019	14.407	11.54
ZnSe	2.431	24.29	19.32
ZnS	2.249	26.40	20.95
PbF2	1.712	35.74	28.02
BaF2	1.454	43.45	33.58
CaF2	1.404	45.42	34.95

**Figure 6:**
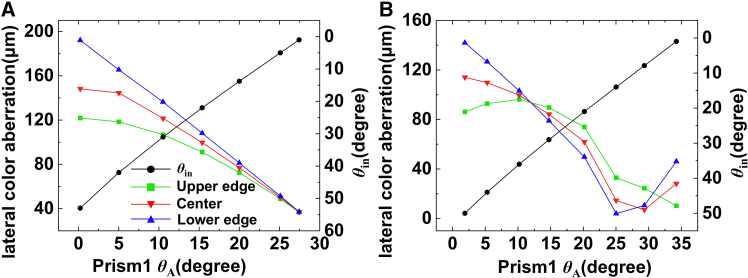
Comparison of lateral color aberrations between two groups of material combinations (A) the same materials, minimum occurs at *θ*_in_ = 1° and (B) different materials, minimum occurs at *θ*_in_ = 7.9°. Except for *θ*_A_, the remaining angles of prism-1 do not affect the light path in the prism.

Achromatic abilities of two combinations of same-material and different-material were analyzed. The right-angle side of prism-2 was set to 30 mm, and the bevel lengths of prism-1 and prism-2 are the same. Values were assigned to *θ*_A_, and iterative optimization was performed according to Equation 3 to obtain the lateral color aberrations of two cases within the band of 3 μm–5 μm, the results are shown in Figure 6. When the materials are the same and *θ*_A_ is 27.4°, the lateral color aberrations at different positions of DMD active area all exceed 36.5 μm, which is much larger than the micromirror size. When the materials are different and *θ*_A_ is 29.3°, the maximum lateral color aberration that is 24.46 μm appears at the upper edge, yet the lateral color aberrations at lower and center areas are less than 7 μm. Therefore, the two prisms were finally designed with the combination of different materials, and their thickness was set to 30 mm. Although the prism design exhibits a certain amount of residual lateral color aberrations, it can be compensated by the illumination lens group to ensure the illumination uniformity of the DMD target surface.

**Figure 7:**
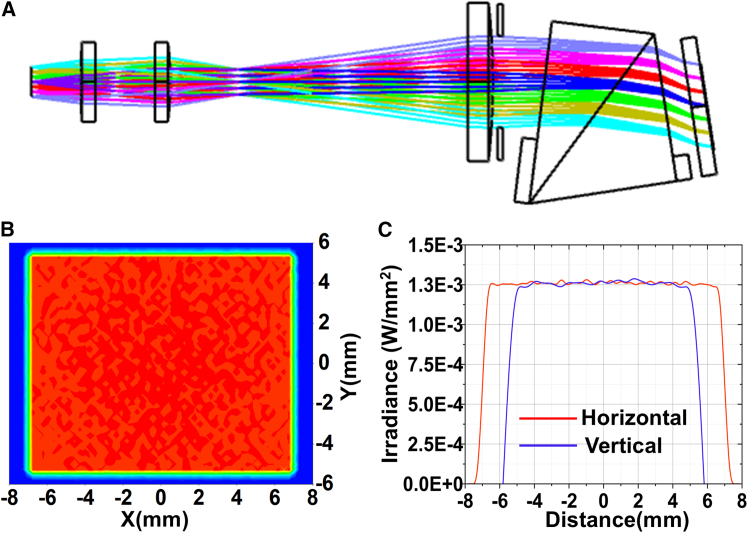
Illumination design results (A) Non-sequential tracking result. (B) Global irradiance distribution. (C) Irradiance distribution in the horizontal and vertical directions.

The illumination design results are shown in Figure 7, and the lens material is Ge. By setting the position and parameters of the rectangular stop reasonably, the illumination spot can be shaped into a rectangle, which prevent stray light generation in the non-active area. When the number of grid divisions is 58 × 42, the irradiance distribution of DMD is shown in Figure 7B and 7C. The uniformity of illumination is better than 94%, and the error estimate is less than 5%. Besides, there is no significant irradiance difference between horizontal and vertical directions.

### Projection optimization

**Figure 8:**
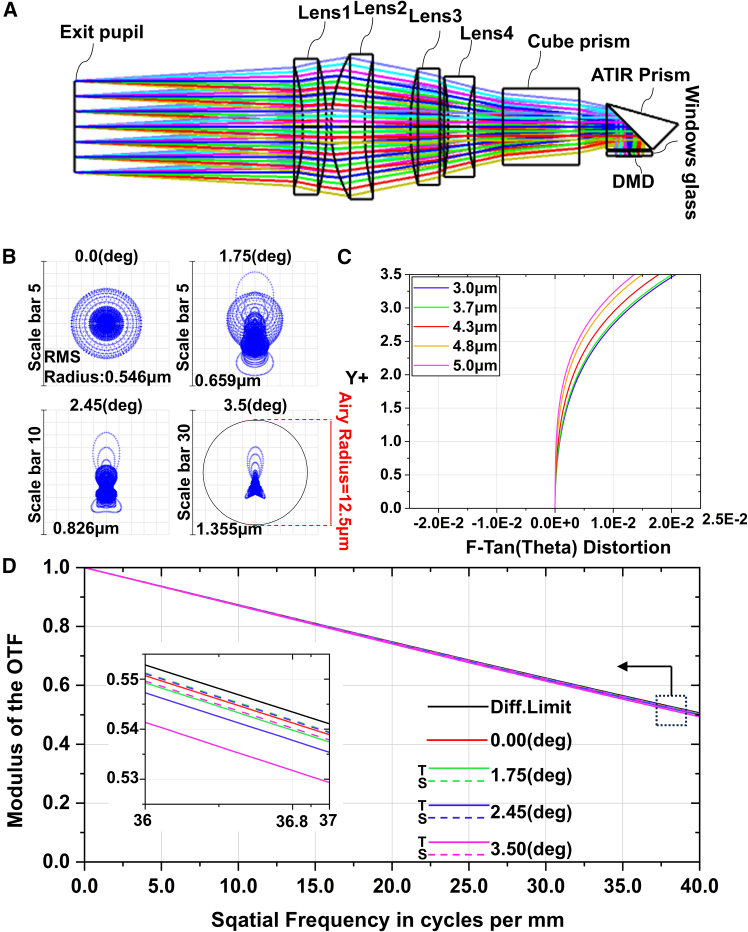
Projection design results (A) Sequential tracing result. (B) Spot diagram. (C) Distortion curves. (D) MTF curves.

The field of view and exit pupil of IRSP should be greater than those of the MWIR camera, and the back working distance is required to be sufficient to set the ATIR prism and cube prism. The optimization result is shown in Figure 8. As can be seen from Figure 8B, the spot diagram of each field of view is located within the Airy spot, and the root-mean-square (RMS) radii of central field and edge field are 0.546 μm and 1.355 μm, respectively. As shown in Figure 8C, the maximum distortion is less than 0.05%. According to the size of the micromirror, the corresponding Nyquist spatial frequency is 36.8 lp/mm. The modulation transfer function (MTF) curves are shown in Figure 8D, and the MTF values of each field of view are greater than 0.5, indicating that the projection quality can meet the use requirements.

Owing to constraints in processing precision, assembly discrepancies, environmental variability, and human errors, the imaging performance of the system frequently falls short of that predicted by software simulations. A tolerance analysis is conducted on the lens, and the tolerance data are show in Table 3.

**Table 3:** Tolerance data

Parameter	Value
Radius/fringe	2
Thickness/mm ±0.02	0.025
Irregularity/fringe	0.25
Tilt(surface)/(′)	0.8
Decenter(surface)/mm	0.008
Tilt(element)/(′)	0.8
Decenter(element)/mm	0.008

Based on the Monte Carlo method, 1000 tolerance analyses are conducted on the system, and the test results are shown in Table 4. At a cutoff frequency of 36.8 lp/mm, over 90% of the MTF values exceed 0.44, indicating satisfactory tolerance performance of the design and ensuring a high production qualification rate during the manufacturing process.

**Table 4:** Tolerance analysis results

Probability	MTF
90%	0.44232101
80%	0.46375895
50%	0.49607396
20%	0.51891657
10%	0.52685428

### Dual optical path calibration

**Figure 9:**
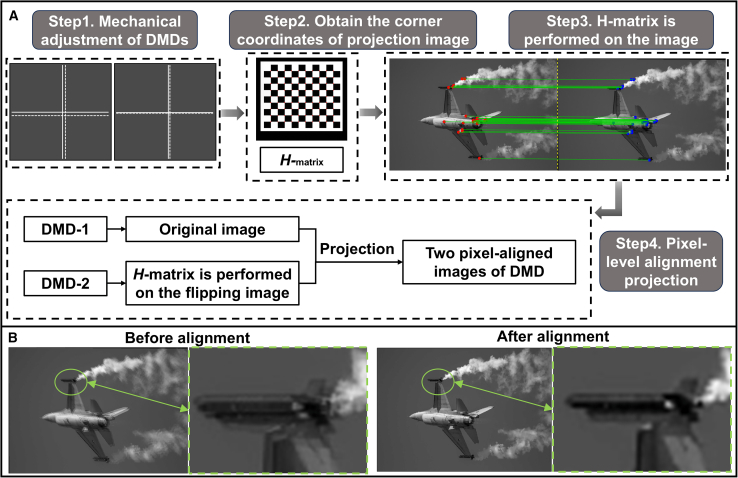
The pixel-level alignment process of two DMDs (A) The calibration process. (B) The simulation projection results before and after alignment.

In the dual-DMD-based infrared optical engine, pixel level alignment is required for the projection images of dual DMDs. Mechanical adjustment alone can cause artifacts in the DMD projection results. Therefore, a projection calibration method based on improved Zhang’s calibration is adopted.^8^^,^^39^ This method places the camera at the exit pupil position of the projection system. The projected images of two DMDs are respectively received by the camera, which can be understood as images captured by the camera from different angles. The mapping relationship between the pixel coordinates of two images can be described by a homography matrix ***H***. The calibration process is shown in Figure 9A. Firstly, two DMDs project same crosshair images and preliminarily adjust the positions of the two DMDs through mechanical adjustment; Secondly, upload the checkerboard image to DMDs and extract all corner coordinates from the two projected images to calculate the matrix ***H***. Then, matrix ***H*** is applied to the original image to obtain the affine transformed image. Finally, due to the odd number of reflections on the optical path, the image uploaded to DMD-2 needs to be flipped. The original image and the image after affine transformation after flipping need to be uploaded to DMD-1 and DMD-2, and projected again to obtain pixel level aligned images. The process of affine transformation can be expressed as

(Equation 6) $${\left[X_{\text{DMD}-2}^{\prime },Y_{\text{DMD}-2}^{\prime },1\right]}^{T}=M^{-1}HM{\left[X_{\text{DMD}-2},Y_{\text{DMD}-2},1\right]}^{T}\text{,}$$

where the right side is the homogeneous coordinates before transformation and the left side is the coordinates, ***M*** is the projection homography mapping transformation matrix. Due to the invertibility of matrix ***M***, matrix ***H*** is similar to ***M***^**−1**^***HM***. This means that the same linear mapping of feature points corresponding to two DMDs is an algebraic expression under different bases. Therefore, matrix ***H*** can be applied to the flipped image of DMD-2 for affine transformation, achieving pixel level alignment between the projected images of DMD-2 and DMD-1. The simulation projection results before and after alignment are shown in Figure 9B, it can be seen that there are artifacts in the projected image and the PSNR is 30.33 dB before alignment, the PSRN of the projected image after alignment is 42.92 dB, indicating that the quality of projected image after alignment is almost the same as that of the original image.

## Discussion

In order to verify IRSP can satisfy the projection requirements for high contrast, a quantitative analysis of the brightness difference in the projected image is conducted. The projection contrast of DMD is defined as the ratio of the radiance in the on-state region to the radiance in the off-state region.

(Equation 7) $$C=\frac{M_{on}}{M_{off}}\text{,}$$

where *M*_on_ and *M*_off_, respectively, represent the maximum and minimum radiation exitance of the on-state and off-state.

### Contrast ratio simulation analysis

**Figure 10:**
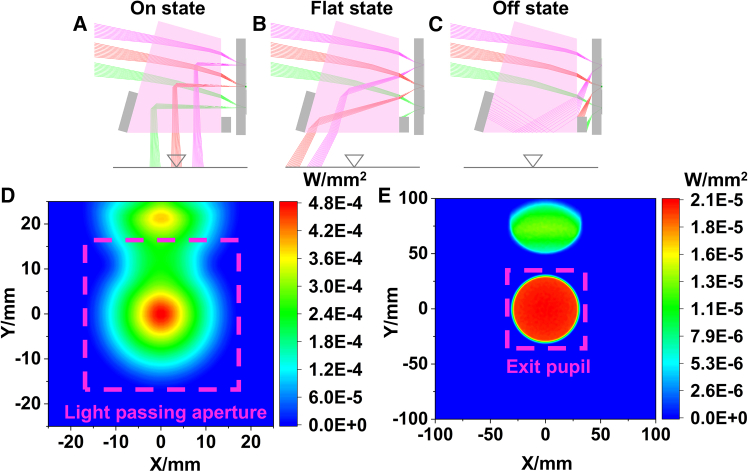
Exit directions corresponding to different states of micromirror (A) on-state, (B) flat-state, and (C) off-state. (D) Irradiance distribution of the incident surface of cube prism. (E) Irradiance distribution of the exit pupil.

The beam exit directions of the micromirror on different states are shown in Figure 10. As can be seen from Figure 10B, when the micromirror is on the flat-state, some beams will enter the projection lens, and the rest are blocked by the baffle. When the micromirror is on the off-state, part of the outgoing beam is totally reflected at the exit surface of prism-2. And all outgoing beams are ultimately blocked by the baffle and cannot enter the projection lens, as shown in Figure 10C.

The incident surface of the cube prism is set as the receiver to track the flat-state and on-state beams. As shown in Figure 10D, the flat-state and on-state spots overlap in the clear aperture region. Then the projection exit pupil is also set as the receiver to continue the non-sequential tracing of the flat and on-state beams. As shown in Figure 10E, the on-state beam fills the entire exit pupil, while the flat-state beam falls outside the exit pupil, and the flat-state spot energy is much lower than that of the on-state spot. Special treatments for inner surface of the barrel, such as processing threads and coating with matte black paint, can eliminate the effect of flat-state beam.

**Figure 11:**
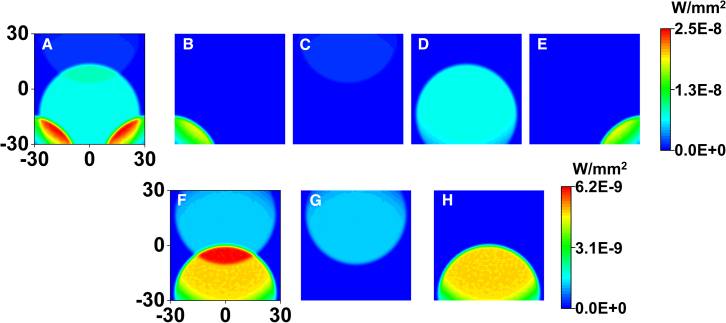
Irradiance distribution at exit pupil for each SM order corresponding to off-state micromirrors (A) 24°, superimposed distribution, (B) (0, −1), (C) (−2, −2), (D) (−1, −1), (E) (−1, 0). (F) 30°, superimposed distribution, (G) (−2, −2), (H) (−1, −1).

In order to verify the influence of diffracted stray light on the optical system, the directions of each diffraction order for the off-state micromirrors are calculated, and the SM beams are non-sequentially traced as shown as Figure 11.

When *θ*_i_ is 24°, the spot superposition of each order at the exit pupil is shown in Figure 11A, and the maximum irradiance is 2.5E−8 W/mm^2^. The diffracted beams of order (0, 1), (−2, −2), (−1, −1), and (−1, 0) can reach the exit pupil surface, and the irradiance distributions are shown in Figures 11B–11E, respectively. When *θ*_i_ is 30°, the spot superposition of each order at the exit pupil is shown in Figure 11F, and the maximum irradiance is 6.2E−9 W/mm^2^, which is reduced by four times. As shown in Figure 10E, the maximum irradiance generated by the on-state beam is 2.1E−5 W/mm^2^ when the illumination incident angle is 30°. According to the calculation results of diffraction efficiency, the maximum irradiance decreases to 1.76E−5 W/mm^2^ when the incidence angle is 24°. Accordingly, we can infer that when the illumination incident angle changes from 24° to 30°, the projection contrast will increase by 4.8 times.

**Figure 12:**
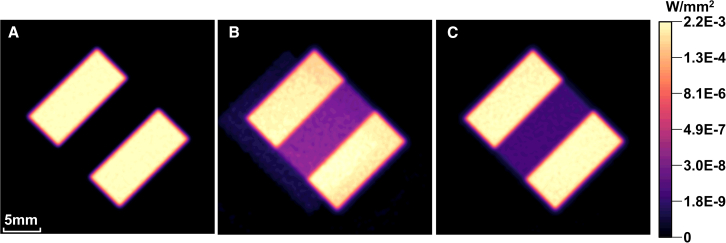
Simulation results of co-aperture infrared optical engine (A) image without diffraction (B) image with diffraction light when *θ*ᵢ = 24° (C) image with diffraction light when *θ*ᵢ = 30°.

Then the white and black stripe image is input into the DMD, and an ideal imaging lens is set at the exit pupil. The obtained projection images under different illumination conditions are shown in Figure 12, where the uniformity of the on-state region in Figure 12A is 94.69%. However, when *θ*_i_ = 24° and considering diffraction light, the uniformity decreases to 87.26% and diffraction induced ghosting appears. As a comparison, when *θ*_i_ becomes 30°, the uniformity improved to 93.5% and the energy distribution is consistent with the non-diffraction case. Diffraction efficiency will rise the overall irradiance distribution of on-state region, and the difference between the above two cases is 10%. It is worth noting that even without considering the improvement of diffraction efficiency, the global contrast of Figure 12C is still more than twice that of Figure 12B.

### Experimental verification

**Figure 13:**
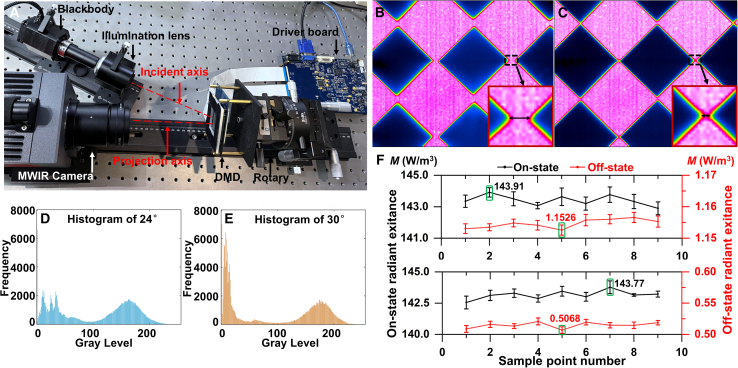
Images received by the thermal imager (A) Experimental platform. (B) *θ*_i_ is 24°. (C) *θ*_i_ is 30°. (D) Histogram for *θ*_i_ = 24°. (E) Histogram for *θ*_i_ = 30° (F) Measurement results: *θ*_i_ = 24° for the upper half and *θ*_i_ = 30° for the lower half. Data at each collection point are the mean of 10 repeated measurements, with error bars representing the distribution range of measurement data at each point. Extreme values are highlighted by rectangular boxes.

After that, a single optical path experimental platform is built, as shown in Figure 13A. The DMD pixel pitch is *d* = 13.68 μm, the tilt of micromirror is ±12°, and the resolution is 1024 × 768. In order to make the diagonal of the micromirror be perpendicular to the plane of the optical platform, the DMD is rotated by 45° around the projection axis using a six-axis rotary. By changing the angle between the illumination axis and the projection axis, a thermal imager is employed to observe the projection energy distribution at different illumination angles. We input the checkerboard image into the DMD, and the captured images are shown in Figures 13B and 13C. By comparing them, the area of the white checkerboard in Figure 13B expands clearly owing to the SM energy, and the definition of the edge contour at the black-and-white junction decreases. While the details in Figure 13C are closer to the original checkerboard image. Moreover, the edge of the checkerboard in the two images presents the rainbow caused by dispersion, which can be weakened by optimizing the optical system. As shown in Figures 13D and 13E, the histogram analysis results show that the gray values of the pixels in Figure 13C mainly concentrate at both ends and the gray values corresponding to the black checkerboards concentrate around 0, indicating that 13 (b) is more severely affected by stray light interference.

To quantitatively describe the contrast of the collected checkerboard image, during the testing process, the blackbody temperature remains constant. Each experiment the DMD target surface needs to be cooled to room temperature (288.17K). The sampling process involves selecting a square area at the center of the infrared camera field of view and sampling the area when it is on-states or off-states. During each sampling, a 3 × 3 grid is uniformly arranged in the area, and a total of 9 points are selected, with the 5th point located at the center of the area. Each sampling point is measured ten times, it can be seen from Figure 13F, when the incident angle is 24°, the contrast is 124.85:1. When the incident angle is adjusted to 30°, the temperature in the off-state region drops to a minimum of 291.36 K, corresponding to a radiation emissivity of 0.5068 W/m^3^, and the contrast increases to 283.68:1, the contrast has increased by about 2.27 times. These results indicate that adjusting the incident angle can effectively reduce diffraction stray light propagating along the projection axis and lower the energy in the off-state region under constant light source temperature. Therefore, it can be proven that the optimized layout of the optical engine can improve the MWIR projection quality indeed.

### Optical efficiency

**Figure 14:**
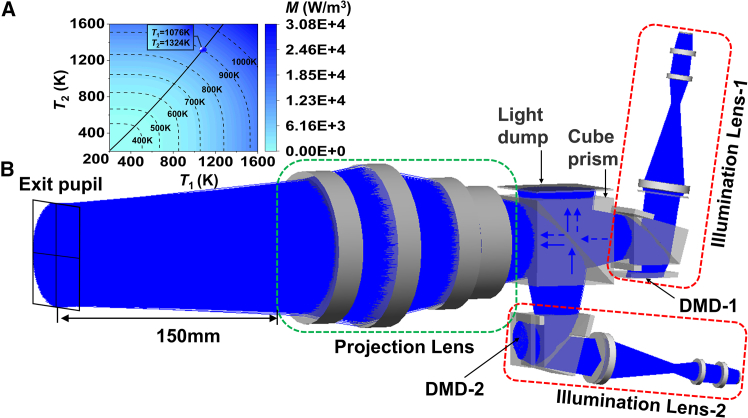
Design result of dual-DMD-based optical engine of IRSP (A) Non-sequential tracking result. (B) Relationship between apparent temperature and blackbody temperatures.

The final design result of dual-DMD based optical engine of IRSP is shown in Figure 14A, and two illumination lenses and one projection lens are combined by a 50/50 cube prism, and a light dump is set above the cube prism to block excess light rays. The solved transmission optical efficiency and the reflective optical efficiency are 16.71% and 17.24%, which are used to calculate the apparent temperature that the optical engine is capable of simulating. The optical efficiency of each component is shown in Table 5.

**Table 5:** Optical efficiency of each component

Optical element	Optical efficiency
Lens	98%
TIR prism	86.8%
DMD	47.2%
Cubic prism transmission part	47%
Cubic prism reflection part	48.5%

According to Planck’s law and the double relationship of radiant exitance between two optical paths, the radiant exitance of IRSP can be obtained by integrating the wavelength range, as.

(Equation 8) $$M=\xi_{Exit\_tra}\cdot \varepsilon \cdot \int_{\lambda_{1}}^{\lambda_{2}}C_{1}\lambda^{-5}\cdot {\left[\exp\left(\frac{c_{2}}{\lambda T_{1}}\right)-1\right]}^{-1}d\lambda +\xi_{Exit\_ref}\cdot \varepsilon \cdot \int_{\lambda_{1}}^{\lambda_{2}}C_{1}\lambda^{-5}\cdot {\left[\exp\left(\frac{c_{2}}{\lambda T_{2}}\right)-1\right]}^{-1}d\lambda \text{,}$$

where *ε* is the effective emissivity set to 0.98, *C*_*1*_ is 3.7418 × 10^−16^ W m^2^, *C*_*2*_ is 1.4388 × 10^−2^ m K, and *T* is blackbody temperature. To ensure that the radiant exitance of two optical paths meets the double relationship, the temperatures of two blackbodies can be calculated, as

(Equation 9) $$\xi_{Exit\_tra}\cdot \varepsilon \cdot \int_{\lambda_{1}}^{\lambda_{2}}C_{1}\lambda^{-5}\cdot {\left[\exp\left(\frac{c_{2}}{\lambda T_{1}}\right)-1\right]}^{-1}d\lambda =2\cdot \xi_{Exit\_ref}\cdot \varepsilon \cdot \int_{\lambda_{1}}^{\lambda_{2}}C_{1}\lambda^{-5}\cdot {\left[\exp\left(\frac{c_{2}}{\lambda T_{2}}\right)-1\right]}^{-1}d\lambda \text{.}$$

The calculated results of radiant exitance of IRSP are shown in Figure 14B. The temperature range of the two blackbodies is set from 200 K to 1600 K. According to Equation 9, the relationship curve that *T*_1_ and *T*_2_ need to satisfy is also exhibited in Figure 14B. Taking the simulated apparent temperature of 900 K as an example, the radiant exitance on the corresponding contour is 1.335E+4 W/m^3^. Only one point satisfies the relationship curve, that is, *T*_1_ = 1082 K and *T*_2_ = 1319 K.

In this paper, through quantitatively calculating the diffraction direction and efficiency of different orders, the optimal illumination incident angle of DMD was derived and realized by the optimized two-element ATIR prism group. Meanwhile, a cube prism was utilized to combine the co-aperture projection lens with two illumination lenses, making up a dual-DMD based optical engine for ultra-high dynamic infrared projection. Furthermore, the diffracted beams of non-PMs under off-state micromirrors are traced, testifying that the local contrast would increase by more than 4 times and the global contrast can still be increased by more than twice even without considering the improvement of diffraction efficiency. And the experimental results are consistent with the simulation. The diffraction analysis-based contrast enhancement method is very helpful with the design of high-dynamic optical systems, which lays an important foundation for the realization of high contrast and high dynamic range infrared projection based on dual DMD.

### Limitations of the study

The long-term stability of common aperture Infrared optical engine under HWIL conditions remains unverified. Especially, the thermal effects in the infrared band induce deformation of the optomechanical structure, thereby reducing contrast. Although this study involves pixel level registration of dual DMDs, they may still be influenced by thermal effects. As precision and stability requirements for HWIL simulation continue to increase, the issue of structural thermal stability will become increasingly critical. To better assess the structural stability of the system, it is necessary to conduct quantitative analysis and investigation of thermal deformation effects.

Model limitations: The diffraction model used in this study is applicable to traditional DMDs based on diagonal flipping mechanism. However, with the popularization and application of the new TRP-DMD in the future, it is necessary to further construct an adapted diffraction model based on its working principle to expand the applicability of this method.

## Resource availability

### Lead contact

Requests for more information and requests for resources, measurement procedures and data, please contact the lead contact, Yue Pan (panyue@cust.edu.cn).

### Materials availability

This study did not generate new unique reagents.

### Data and code availability

• All data reported in this paper will be shared by the lead contact upon request.
• All original code has been deposited at Zenodo and is publicly available at doi https://doi.org/10.5281/zenodo.19935282 as of the date of publication.
• Any additional information required to reanalyze the data reported in this paper is available from the lead contact upon request.

## Acknowledgments

This work was supported by the 10.13039/100015800Jilin Province Development and Reform Commission (2024C007-1), the 10.13039/501100002858China Postdoctoral Science Foundation (2025M770826), 10.13039/501100001809National Natural Science Foundation of China (61903048).

## Author contributions

Conceptualization, Y.P.; methodology, Y.P. and L.X.; investigation, Z.W. and Y.Z.; formal analysis, Z.W.; data curation, Z.W., L.X., and Y.Z.; software, Y.Z.; validation, M.H.; visualization, S.S.; writing – original draft, Z.W.; writing – review and editing, Y.P.; project administration, X.X.; funding acquisition, X.X.; supervision, Y.P.

## Declaration of interests

The authors declare no competing interests.

## STAR★Methods

### Key resources table

**Table:** 

REAGENT or RESOURCE	SOURCE	IDENTIFIER
**Software and algorithms**		

Ansys Zemax OpticStudio	Zemax	Ansys Zemax OpticStudio | Optical Design and Analysis Software
LightTools	Synopsys, Inc	LightTools Illumination Design Software | Keysight
Diffraction calculation	Zenodo	Doi: https://doi.org/10.5281/zenodo.19935282

**Other**		

MWIR Camera	InfraTec	https://www.infratec.cn/thermography/
DMD	CAS MICROSTAR OPTOELECTRONIC TECHNOLOGY CO.	https://www.casmicrostar.com/
Blackbody	Beijing AOEO technology Co.,Ltd.	http://aoeo.com.cn/Product_View/?19-56-149.html

### Experimental model and study participant details

Not applicable. This study is an optical engineering research focusing on the design and experimental verification of a co-aperture infrared optical engine, and does not involve any biological experimental models or human/animal participants.

### Method details

Modeling and experimental verification are mainly carried out using LightTools optical software and Zemax. The lighting and projection lenses were initially designed in Zemax to meet specified optical requirements, and the resulting models were then imported into LightTools for integrated system analysis.

By analyzing the relationship between diffraction efficiency and angle, particularly for the PM and SM. Based on this analysis, the optimal illumination incident angle was determined to minimize the diffraction beam entering the exit pupil as much as possible, otherwise it would reduce the projection contrast. Using Snell’s law, key parameters and geometric structures of TIR prisms were designed to achieve the desired illumination angle.

Ray-tracing simulations were performed in LightTools to evaluate the beam separation capability of the designed TIR prism for both on-state and off-state DMD micromirrors. Through these simulations, the optimal position of the baffle was determined to effectively block stray light originating from flat-state and off-state micromirrors.

An important consideration when applying the diffraction model is that calculated results must be constrained to the interval from −90° to 90°. Without this restriction, the solution exhibits a periodic cycle with a π interval. All stray-light analyses related to diffraction were based on results from this model and carried out within LightTools. Specifically, the optimized optical engine was imported into LightTools, and the DMD surface was treated as an area light source. Each diffraction order was assigned an energy value corresponding to its calculated diffraction efficiency, and the propagation direction was set according to the azimuth and diffraction elevation angles derived from the model. Non-sequential ray tracing provided the irradiance distribution at the exit pupil plane, with final results presented in Figure 11.

It is worth noting that material properties assigned to optical elements in Zemax do not automatically transfer when the model is imported into LightTools. These properties must be redefined and verified manually within LightTools. Furthermore, the irregular prism geometry required for this design cannot be built directly in Zemax. As a workaround, the prism model was exported as a POB file that a 3D format defined by vertex coordinates, and its structure and coordinates were then reconstructed and calibrated in LightTools. The ready-to-use POB files for the two prisms, labeled ATIR1 and ATIR2, are provided with this work.

**Table:** 

Continued	
Nomenclature and units	
*C*	Projection contrast ratio [/]
*L*_on_	Radiance of the DMD on-state region [W/(m^2^·sr)]
*L*_off_	Radiance of the DMD off-state region [W/(m^2^·sr)]
*θ*_*i*_	Incident elevation angle [°]
*φ*_*i*_	Incident azimuth angle [°]
*θ*_*d*_	Diffraction elevation angle [°]
*φ*_*d*_	Diffraction azimuth angle [°]
*λ*	Operating wavelength [μm]
*d*	DMD micromirror pixel pitch [μm]
*(p,q)*	Diffraction order [/]
*η*	Diffraction efficiency [/]
*ξ*	Spatial frequency shift caused by the flipped micromirror [lp/mm]
*γ*	Tilt angle of DMD micromirror [°]
*θ*_*A*_	Apex angle of Prism 1 [°]
*θ*_*in*_	Ray incident angle at the entrance surface of Prism 1 [°]
*n*_*1*_	Refractive index of Prism 1 material [/]
*n*_*2*_	Refractive index of Prism 2 material [/]
*θ*_*3*_	Ray incident angle at the exit surface of Prism 1 [°]
*θ*_*DMD*_	Incident elevation angle on DMD active area [°]
*F/#*	F-number of the optical system [/]
*RMS*	Root-mean-square radius of the focused spot [μm]
*MTF*	Modulation transfer function [/]
*PSNR*	Peak signal-to-noise ratio [dB]
*M*_*on*_	Maximum radiant exitance of the on-state region [W/m^2^]
*M*_*off*_	Minimum radiant exitance of the off-state region [W/m^2^]
*ε*	Effective emissivity [/]
*C*_*1*_	First radiation constant [W·m^2^]
*C*_*2*_	Second radiation constant [m·K]
*T*	Blackbody thermodynamic temperature [K]
*T*_*1*_	Blackbody temperature of DMD-1 channel [K]
*T*_*2*_	Blackbody temperature of DMD-2 channel [K]
*M*	Radiant exitance [W/m^2^]
*H*	Homography matrix for dual-DMD projection image pixel alignment [/]

### Quantification and statistical analysis

Numerical simulation, image processing and statistical quantification in this study were conducted using MATLAB or Python. Diffraction results presented in Figure 1, Figures 2 and 3 were numerically computed from Equations 1 and 2 via the provided MATLAB scripts, and all simulation data are fully reproducible. Experimental infrared images under variable illumination angles were acquired using the optical setup shown in Figure 13A. Statistical analysis of image grayscale distributions was performed to obtain results in Figures 13D and 13E. For quantitative energy analysis, 10 independent repeated measurements were conducted under the same experimental conditions. The statistical data in Figure 13F uses 9 spatial sampling points, with the 5th point designated as the center sampling position. Arithmetic mean values of the 10 repeated measurements were adopted to describe the central tendency of measured energy data and error bars for different sampling points can be derived from these ten measurements.

### Additional resources

The MATLAB code for calculating the diffraction characteristics of Digital Micromirror Devices (DMDs) is available at the following link. This code implements the grating diffraction equations presented in this work and generates the data corresponding to Figures 1, 2, and 3. It calculates the relationship between incidence angle, incidence azimuth angle, diffraction azimuth angle, diffraction elevation angle, and diffraction efficiency, with adjustable parameters including micromirror size, tilt angle, wavelength, and diffraction order. Code Source: https://doi.org/10.5281/zenodo.19935282.
